# Mitochondria selective *S*-nitrosation by mitochondria-targeted *S*-nitrosothiol protects against post-infarct heart failure in mouse hearts

**DOI:** 10.1002/ejhf.100

**Published:** 2014-05-31

**Authors:** Carmen Methner, Edward T Chouchani, Guido Buonincontri, Victoria R Pell, Stephen J Sawiak, Michael P Murphy, Thomas Krieg

**Affiliations:** 1 Department of Medicine, University of Cambridge, Addenbrooke's HospitalCambridge, UK; 2 MRC Mitochondrial Biology UnitCambridge, UK; 3 Wolfson Brain Imaging Centre, University of Cambridge, Addenbrooke's HospitalCambridge, UK

**Keywords:** Myocardial infarction, Heart failure, Nitric oxide, Free radicals, Magnetic resonance imaging

## Abstract

**Aims:**

Recently it has been shown that the mitochondria-targeted *S*-nitrosothiol MitoSNO protects against acute ischaemia/reperfusion (IR) injury by inhibiting the reactivation of mitochondrial complex I in the first minutes of reperfusion of ischaemic tissue, thereby preventing free radical formation that underlies IR injury. However, it remains unclear how this transient inhibition of mitochondrial complex I-mediated free radicals at reperfusion affects the long-term recovery of the heart following IR injury. Here we determined whether the acute protection by MitoSNO at reperfusion prevented the subsequent development of post-myocardial infarction heart failure.

**Methods and results:**

Mice were subjected to 30 min left coronary artery occlusion followed by reperfusion and recovery over 28 days. MitoSNO (100 ng/kg) was applied 5 min before the onset of reperfusion followed by 20 min infusion (1 ng/kg/min). Infarct size and cardiac function were measured by magnetic resonance imaging (MRI) 24 h after infarction. MitoSNO-treated mice exhibited reduced infarct size and preserved function. In addition, MitoSNO at reperfusion improved outcome measures 28 days post-IR, including preserved systolic function (63.7 ±1.8% LVEF vs. 53.7 ± 2.1% in controls, *P* = 0.01) and tissue fibrosis.

**Conclusions:**

MitoSNO action acutely at reperfusion reduces infarct size and protects from post-myocardial infarction heart failure. Therefore, targeted inhibition of mitochondrial complex I in the first minutes of reperfusion by MitoSNO is a rational therapeutic strategy for preventing subsequent heart failure in patients undergoing IR injury.

## Introduction

During myocardial infarction (MI), mitochondria are central to mediating the damage that underlies ischaemia/reperfusion (IR) injury.^1^–^3^ In particular, mitochondrial reactive oxygen species (ROS) production in conjunction with mitochondrial matrix Ca^2+^ overload converge to induce opening of the mitochondrial permeability transition pore, which is thought to drive a significant amount of acute damage.^4^ Therefore, decreasing ROS production by mitochondria upon reperfusion is an appealing therapeutic strategy to minimize IR injury.

Among the agents that protect against IR injury are nitric oxide (NO) and its metabolites,^5^–^8^ but clinical use has been limited by their pleiotropic action and uncertainty regarding their cardioprotective targets and mechanism(s). However, recently we developed a mitochondria-targeted *S*-nitrosothiol (MitoSNO) to elucidate a critical cardioprotective mechanism mediated specifically by mitochondrial *S*-nitrosation.^9^,^10^

We identified an essential component of MitoSNO-mediated protection to be due to the reversible *S*-nitrosation of a critical functional cysteine, Cys39 on the ND3 subunit of mitochondrial complex I, which only becomes susceptible to modification following prolonged ischaemia.^11^ This transient modification inhibits rapid reactivation of mitochondrial complex I during reperfusion, thereby preventing mitochondrial ROS production. Through this acutely acting mechanism, MitoSNO systemically prevented acute cardiac IR injury. This suggested that MitoSNO could be used to decrease cardiac IR injury in a range of pathological situations, as it is very rapidly taken up from the circulation into heart mitochondria upon i.v. administration during the reperfusion phase of IR injury, does not affect non-ischaemic tissue, and its inhibition of complex I is rapidly reversed after a few minutes of reperfusion. These effects were in stark contrast to induction of non-mitochondrial *S*-nitrosation, which has been shown to be insufficient to mitigate mitochondrial ROS and protect against reperfusion injury *in vivo*.^11^ However, to be a useful therapeutic strategy, the selective *S*-nitrosation of complex I must lead to an improved prognosis for the patient by decreasing the amount of long-term cardiac tissue damage and organ dysfunction that are frequent consequences of an IR injury and which lead on to chronic heart disease.

Therefore, the aim of this study was to determine whether the acute and transient action of MitoSNO during reperfusion of the ischaemic heart would protect against the chronic and persistent organ damage that leads to post-MI heart disease and failure. We hypothesized that the burst of ROS emanating from the rapid reactivation of mitochondrial complex I during reperfusion drives the initial acute damage to the heart that leads over time to the development of subsequent heart failure. Therefore, prevention of acute ROS production by complex I during reperfusion by MitoSNO would not only decrease acute IR injury but would also protect the heart from post-MI heart damage and dysfunction measured weeks after the initial MI.

## Methods

### *In vivo* mouse model of myocardial infarction

Infarct size following IR in an *in situ* open chest mouse model was measured as previously described.^12^

### Magnetic resonance imaging

Cardiac late gadolinium-enhanced magnetic resonance imaging (LGE-MRI) and cine MRI were performed as recently described.^12^

## Results and Discussion

### Protection against cardiac ischaemia/reperfusion injury by administration of mitochondria-targeted *S*-nitrosothiol at reperfusion

We have recently demonstrated that administration of the mitochondria-selective *S*-nitrosating agent MitoSNO decreases acute cardiac IR injury by *S*-nitrosation of proteins within mitochondria, and that this protection is primarily due to the functional *S*-nitrosation of Cys39 on the ND3 subunit of complex I that decreases mitochondrial superoxide production upon reperfusion.^11^ However, for this approach to be useful clinically it is important to show that the acute protective action of MitoSNO extended into the long term.

**Figure 1:**
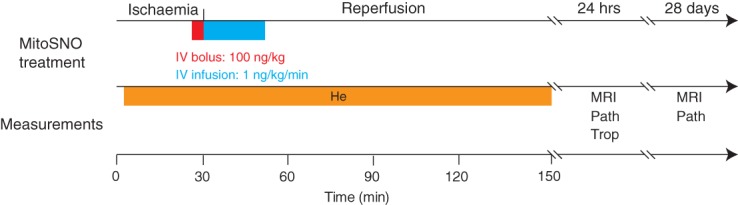
Schematic diagram of experimental protocols and measurements. Summary of the mitochondria-targeted *S*-nitrosothiol (MitoSNO) treatment protocol applied to the mouse LAD occlusion model as well as timing and the parameters measured. He, haemodynamic parameters; MRI, magnetic resonance imaging; Path, time of sacrifice for histopathology; Trop, troponin.

An important observation from our previous work was that MitoSNO is only effective when it is perfused into ischaemic tissue, as protective inhibition of complex I by *S*-nitrosation of ND3 Cys39 can only be achieved in this setting.^11^ As it is well established that reperfusion of ischaemic tissue in the clinic leads to variable rates of reintroduction of blood supply, we modified our treatment protocol combining a bolus injection with persistent perfusion over 20 min to ensure delivery of active MitoSNO to all parts of the tissue as reperfusion progressed. This protocol is summarized in *Figure* 1: mice were subjected to 30 min LAD occlusion, and then administered MitoSNO as a systemic i.v. bolus followed by continuous infusion beginning 5 min before reperfusion. A series of measures of heart function were applied over the subsequent 4 weeks to assess whether this treatment improved the long-term recovery of the heart.

### Mitochondria-targeted *S*-nitrosothiol at reperfusion reduces infarct size and troponin release assessed 24 h post-myocardial infarction

**Figure 2:**
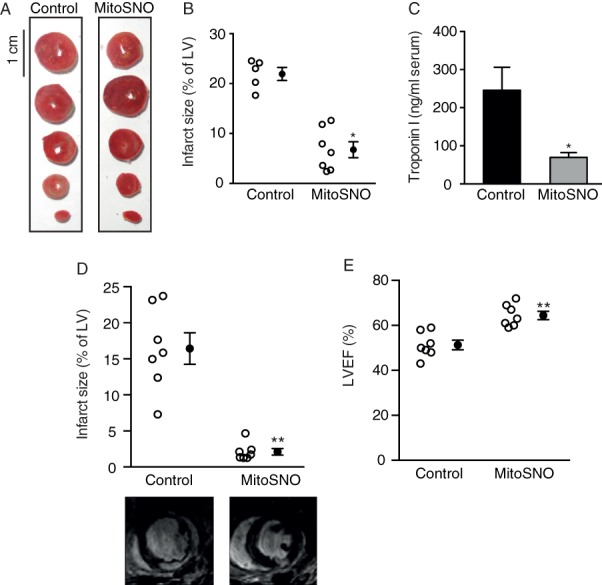
Effects of mitochondria-targeted *S*-nitrosothiol (MitoSNO) on infarct size and heart function 24 h post-myocadial infarction (MI). (*A*) Representative heart cross-sections for infarct size assessment via histological 2,3,5-triphenyltetrazolium chloride (TTC) staining. (*B*) Quantification of TTC-stained infarcts. (*C*) Quantification of troponin I level in blood serum mirrored 24 h post-MI. (*D*) The infarct size quantification by late gadolinium-enhanced magnetic resonance imaging (LGE-MRI) 24 h post-MI for the MitoSNO-treated group compared with the untreated control group including representative images. (*E*) Assessment of LVEF 24 h post-MI. ^**^P < 0.001; ^*^P < 0.05.

We first assessed whether this MitoSNO administration protocol protected against heart tissue damage measured by 2,3,5-triphenyltetrazolium chloride (TTC) staining 24 h post-reperfusion (*Figure* 2*A*). This showed that a systemic i.v. bolus and infusion of MitoSNO beginning 5 min before reperfusion resulted in a significant reduction in infarct size (*Figure* 2*B*) as well as significantly lower values of the clinically used marker, serum troponin I, 24 h post-MI in the MitoSNO-treated group (*Figure* 2*C*).

In addition, cardiac LGE-MRI 24 h post-MI confirmed drastically reduced infarct size in the mice subjected to acute MitoSNO treatment (*Figure* 2*D*; Supplementary material online, *Videos S1a* and *S2a*), as well as increased post-MI LV function (*Figure* 2*E*). Numerical data of MRI analyses are provided in Supplementary material online, *Table S1*. These findings indicate that MitoSNO affords robust protection against IR injury as assessed by clinically relevant measures 24 h post-MI.

### Lack of effect of mitochondria-targeted *S*-nitrosothiol on haemodynamic parameters

**Figure 3:**
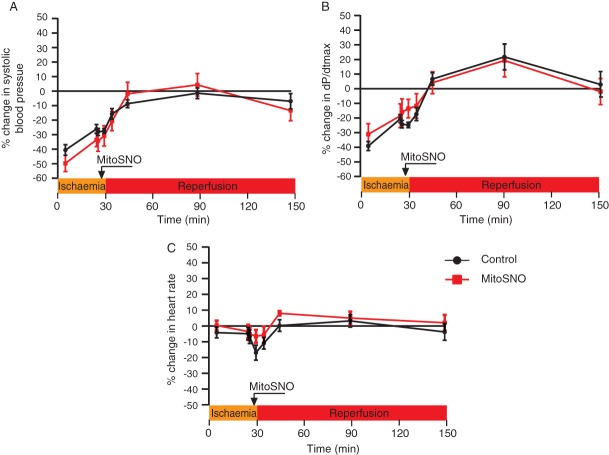
Haemodynamic effect of mitochondria-targeted *S*-nitrosothiol (MitoSNO). LV catheterization was used to obtain blood pressure data during infarct size measurement in the acute model of 30 min ischaemia followed by 2 h reperfusion, comparing MitoSNO treatment with controls. Haemodynamic parameters: (*A*) systolic blood pressure; (*B*) d*P*/d*t*max; and (*C*) heart rate were determined and plotted as changes to baseline. *n* = 7.

A concern in the use of all NO-based drugs is that release of free NO may result in alterations to blood pressure or haemodynamics that can preclude their clinical use. This should not be a concern with MitoSNO as it is rapidly taken up into mitochondria within tissues where it transfers a nitrosonium moiety directly to mitochondrial thiols, with negligible release of free NO at therapeutic concentrations.^11^,^13^ In addition, we have recently demonstrated that MitoSNO acts independently of non-mitochondrial cGMP pathways, and that an acute dose of MitoSNO has no effect on haemodynamics in a normally respiring heart.^11^,^13^ However, the possibility remains that the administration of MitoSNO during IR injury may alter haemodynamics, in addition to the possibility that continuous infusion of MitoSNO may disrupt haemodynamics while an acute bolus alone did not. To assess both of these possibilities, blood pressure and heart function measurements were taken at all stages of acute MI and subsequent reperfusion via LV catheterization, and the effects of MitoSNO administration and infusion on these were assessed for up to 2 h post-reperfusion (*Figure* 3). We observed no difference in systolic blood pressure between the MitoSNO-treated and vehicle-treated groups (*Figure* 3*A*). In addition, MitoSNO also had no effect on d*P*/d*t*max (*Figure* 3*B*) or heart rate (*Figure* 3*C*). Together, these data confirmed that a dosing regimen of MitoSNO that was robustly cardioprotective and which included continual infusion for 20 min had no effect on haemodynamics or blood pressure. Therefore, MitoSNO can be administered as a bolus followed by continual infusion systemically at cardioprotective levels without affecting haemodynamics, which avoids a major limitation that restricts the use of other cardioprotective NO donors.

### Mitochondria-targeted *S*-nitrosothiol given at reperfusion protects from long-term post-myocardial infarction heart failure and myocardial fibrosis

The above data show that this dosing regimen of MitoSNO protects heart function against IR damage when measured 24 h after reperfusion. However, a common consequence of IR injury in MI is the subsequent long-term development of heart dysfunction and damage, and it was unknown whether MitoSNO—which acts only in the first minutes of reperfusion—could impact on these long-term sequelae. To see if MitoSNO treatment at reperfusion afforded long-term protection, we assessed how well heart damage resolved over 28 days.

**Figure 4:**
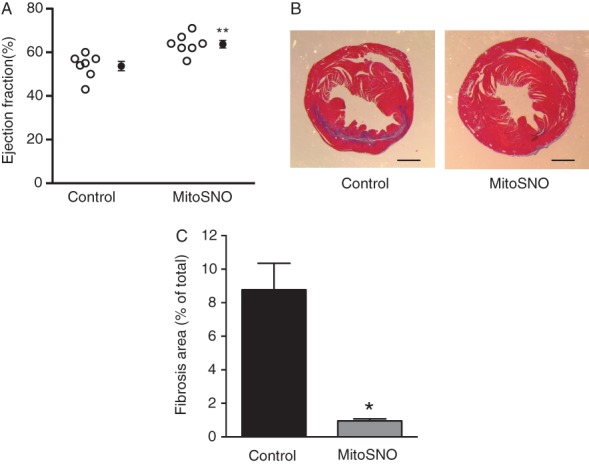
Heart function and damage measured 28 days post-myocardial infarction (MI). (*A*) Assessment of LVEF 28 days post-MI. (*B*) Representative heart slices after Masson trichrome staining of control and mitochondria-targeted *S*-nitrosothiol (MitoSNO)-treated hearts. (*C*) Quantification of collagen content in total heart comparing MitoSNO and control hearts. ^*^P < 0.01; ^**^P < 0.001.

Determination of LVEF indicated significant improvement in the heart function of MitoSNO-treated mice compared with controls 28 days post-MI (*Figure* 4*A*; Supplementary material online, *Videos S1b* and *S2b*). In addition, measurement of tissue fibrotic remodelling 28 days post-MI showed a significant decrease in collagen content in the MitoSNO-treated hearts compared with the control hearts (*Figure* 4*B* and *C*). Therefore, acute administration of MitoSNO upon reperfusion not only decreased acute heart damage but also led to an improved long-term functional outcome for the heart. These results are particularly striking in the context of our recent characterization of the kinetics of MitoSNO activity at reperfusion *in vivo*.^11^ Intravenous administration of MitoSNO results in mitochondrial *S*-nitrosation activity only in the first minutes of reperfusion, which is essential and necessary to mitigate mitochondrial ROS through mitochondrial complex I, and consequent initiation of acute cell death.^11^ With the findings described herein, this transient MitoSNO activity is clearly also critical for long-term recovery of the heart post-reperfusion.

## Conclusion

Although recent years have seen an increase in survival after an acute MI, the long-term implications of cardiac IR injury with the development of chronic heart failure remain. Here we find MitoSNO to be a highly promising and clinically applicable cardioprotective therapy, which could be applied acutely after an infarct and would protect the heart not only against acute damage but also against long-term injury. Furthermore, with previous elucidation of the mechanism of MitoSNO action, here we demonstrate that transient MitoSNO action in the first minutes of reperfusion is sufficient to protect from long-term post-MI sequelae. Additionally, since MitoSNO delivers NO highly selectively within mitochondria, the hindering side effects of generalized NO therapy are not present, facilitating the translation into vulnerable MI patients.

## Supporting Information

**Appendix S1.** Detailed Methods, *In vivo* mouse model of myocardial infarction, MRI, Histological staining for fibrosis, Statistics

**Table S1.** MRI-derived left ventricular volumes at the acute stage (24 h post-MI), data are mean ± SEM. ^*^p < 0.05, ^**^p < 0.01, ^***^p < 0.001

**Table S2.** MRI-derived left ventricular volumes at the chronic stage (28 days post-MI), data are mean ± SEM. ^*^p < 0.05, ^**^p < 0.01

**Video S1.** Representative MRI videos of control heart 24 h (a) and 28 days (b) after ischaemic injury in four-chamber view.

**Video S2.** Representative MRI videos of MitoSNO-treated heart 24 h (a) and 28 days (b) after ischaemic injury in four-chamber view.
