# Supplementary material for: Mitochondria selective S-nitrosation by mitochondria-targeted S-nitrosothiol protects against post-infarct heart failure in mouse hearts
Source: Eur J Heart Fail. 2014 May 31;16(7):712–7. doi: 10.1002/ejhf.100 (PMC4231226; doi:10.1002/ejhf.100)
Supplement: Supplementary file 1 — Appendix S1. Detailed Methods, In vivo mouse model of myocardial infarction, MRI, Histological staining for fibrosis, Statistics [file ejhf0016-0712-sd1.doc]

**SUPPLEMENTAL MATERIAL**

**Mitochondria selective *S*-nitrosation by MitoSNO protects against post-infarct heart failure in mouse hearts**

Carmen Methner PhD^1^, Edward T. Chouchani PhD^1,2^, Guido Buonincontri^3^, Victoria R. Pell^1^, Stephen J. Sawiak PhD^3^, Michael P. Murphy PhD^2^, Thomas Krieg MD PhD^1,*^

^1^Department of Medicine, University of Cambridge, Addenbrooke's Hospital, Hills Road, Cambridge, CB2 2QQ, UK.

^2^ MRC Mitochondrial Biology Unit, Hills Road, Cambridge CB2 0XY, UK.

^3^ Wolfson Brain Imaging Centre, University of Cambridge, Addenbrooke’s Hospital, Hills Road, CB2 0QQ, UK

*To whom correspondence should be addressed:

Phone: (+44) 1223 762584
Email: tk382@medschl.cam.ac.uk

**Detailed Methods**

All procedures were conducted in accordance with the Animals (Scientific Procedures) Act 1986 (PPL 80/2393) and the University of Cambridge Policy on the Use of Animals in Scientific Research and approved by the Home Office (UK) Animals Scientific Procedures Department (ASPD).

***In vivo* mouse model of myocardial infarction**

Infarct size following ischemia/reperfusion in an *in situ* open chest mouse model was measured as previously described[^1^](#_ENREF_1). Male C57BL/6 mice were used (Charles River Laboratories, UK). All animals were subjected to 30 min occlusion of a main branch of the left anterior descending (LAD) coronary artery followed by either 24 h or 28 days reperfusion. Mice received either intravenous saline or 100 ng/kg MitoSNO 5 min before the onset of reperfusion followed by 20 min infusion (1 ng/kg/min).

At the end of each experiment used for the 24 h end point cardiac Troponin I was measured in blood serum taken prior to heart removal. In order to determine the development of post-MI heart failure, mice underwent late-gadolinium enhanced (LGE) magnetic resonance imaging (MRI) after 24 h for infarct size and functional measurements, as well as functional MRI after 28 days. After 28 days the heart was removed and prepared for histological analysis.

**MRI**

Cardiac late gadolinium enhanced magnetic resonance imaging (LGE-MRI) and magnetic resonance imaging (MRI) were performed as recently described[^1^](#_ENREF_1). Briefly, animals were anaesthetized with gaseous isoflurane, gating of the MRI sequences was achieved with ECG monitoring, and body temperature was monitored and kept constant at 37°. LGE-MRI was performed 24 hours after IR injury at 4.7 T with a Bruker BioSpec 47/40 system (Bruker Inc., Ettlingen, Germany) while the MRI protocol excluding LGE was repeated 28 days after IR injury. Numerical data of MRI analyses are provided in Supplementary Table 1. The investigator performing the LGE-MRI analyses was blinded to the intervention status of each mouse.

For image analysis the papillary muscles and trabeculations were excluded from the delineation of the LV at each phase of the cardiac cycle. The regions from each slice were combined using Simpson’s rule to provide LV mass, end diastolic volume, end systolic volume, stroke volume and ejection fraction using Segment v1.9. Infarcts were manually delineated on the LGE images. Values are expressed as ratios of the LV mass as measured from the standard cine protocol.

**Histological staining for fibrosis**

After 28 days the hearts were excised, stored over night in 10% formalin and embedded in paraffin before 9 mircons thick section were taken throughout the heart. Masson’s Trichrome staining was performed according to the manual of the agent Kit form Sigma (HT15, St. Louis, MO, USA). Briefly, slides were deparaffinized in deionized water and fixed in Bouin’s solution. After washing and incubation in Weigert’s Irono Heamatoxylin solution, the slides were incubated in Biebrich Scarlet Acid Fuchsin and Anilin Blue solution for staining. Finally the slides were dehydrated in ethanol and mounted. The nuclei were stained black, the collagen fibers blue and muscles red. The amount of collagen in total area of heart was quantified by using Photoshop. The investigator performing histological analysis was blinded to the intervention status of each mouse heart.

**Statistics**

All data are presented as mean ± standard error of the mean (SEM). The infarct size is shown as percentage of area at risk. The blood pressure values are plotted as percentage of the respective baselines. Differences among groups were compared by one-way ANOVA with Turkey’s post hoc test. A value of p<0.05 was considered significant.

**Supplemental Tables**

|  | Controls (n=7) | MitoSNO (n=7) |
| --- | --- | --- |
| LVM (µl) | 104 ± 4 | 88 ± 5 * |
| LVEDV (µl) | 65 ± 6 | 49 ± 4 * |
| LVESV (µl) | 32 ±3 | 18 ±2 ** |
| LVSV (µl) | 34 ± 3 | 31 ± 2 |
| LVEF (%) | 51 ± 2 | 64 ± 2 *** |
| Infarct size (% LV) | 16 ± 2 | 2.1 ± 0.5*** |

**Supplementary Table 1: MRI-derived left ventricular volumes at the acute stage (24 h post-MI), data are mean ± SEM. * p <0.05, ** p<0.01, *** p<0.001**

|  | Controls (n=7) | MitoSNO (n=7) |
| --- | --- | --- |
| LVM (µl) | 120 ± 7 | 101 ± 6 * |
| LVEDV (µl) | 76 ± 6 | 60 ± 6 * |
| LVESV (µl) | 36 ± 4 | 22 ± 3 * |
| LVSV (µl) | 40 ± 3 | 38 ± 3 |
| LVEF (%) | 54 ±2 | 64 ±1 ** |

**Supplementary Table 2:** MRI-derived left ventricular volumes at the chronic stage (28 days post-MI), data are mean ± SEM. * p <0.05, ** p<0.01

**Legends for Video Files**

**Supplementary Videos 1:** Representative MRI videos of control heart 24 h **(a)** and 28 days **(b)** after ischemic injury in 4-chamber view.

**Supplementary Videos 2:** Representative MRI videos of MitoSNO-treated heart 24 h **(a)** and 28 days **(b)** after ischemic injury in 4-chamber view.

**Supplementary References**

1. Buonincontri G, Methner C, Krieg T, Carpenter TA, Sawiak SJ. A fast protocol for infarct quantification in mice. *J Magn Reson Imaging*. 2013;38:468-473.
