# Supplementary material for: Mitochondria selective S-nitrosation by mitochondria-targeted S-nitrosothiol protects against post-infarct heart failure in mouse hearts
Source: Eur J Heart Fail. 2014 May 31;16(7):712–7. doi: 10.1002/ejhf.100 (PMC4231226; doi:10.1002/ejhf.100)
Supplement: Supplementary file 2 — Table S1. MRI-derived left ventricular volumes at the acute stage (24 h post-MI), data are mean ± SEM. *p < 0.05, **p < 0.01, ***p < 0.001 [file ejhf0016-0712-sd2.doc]

**Supplementary Table 1:** MRI-derived left ventricular volumes at the acute stage (24 h post-MI), data are mean ± SEM. * p <0.05, ** p<0.01, *** p<0.001

|  | Controls (n=7) | MitoSNO (n=7) |
| --- | --- | --- |
| LVM (µl) | 104 ± 4 | 88 ± 5 * |
| LVEDV (µl) | 65 ± 6 | 49 ± 4 * |
| LVESV (µl) | 32 ±3 | 18 ±2 ** |
| LVSV (µl) | 34 ± 3 | 31 ± 2 |
| LVEF (%) | 51 ± 2 | 64 ± 2 *** |
| Infarct size (% LV) | 16 ± 2 | 2.1 ± 0.5*** |

|  |  |  |
| --- | --- | --- |
|  |  |  |
|  |  |  |
|  |  |  |
|  |  |  |
|  |  |  |
