# Supplementary material for: Mitochondria selective S-nitrosation by mitochondria-targeted S-nitrosothiol protects against post-infarct heart failure in mouse hearts
Source: Eur J Heart Fail. 2014 May 31;16(7):712–7. doi: 10.1002/ejhf.100 (PMC4231226; doi:10.1002/ejhf.100)
Supplement: Supplementary file 3 — Table S2. MRI-derived left ventricular volumes at the chronic stage (28 days post-MI), data are mean ± SEM. *p < 0.05, **p < 0.01 [file ejhf0016-0712-sd3.doc]

|  |  |  |
| --- | --- | --- |
|  |  |  |
|  |  |  |
|  |  |  |
|  |  |  |
|  |  |  |
|  |  |  |

**Supplementary Table 2:** MRI-derived left ventricular volumes at the chronic stage (28 days post-MI), data are mean ± SEM. * p <0.05, ** p<0.01

|  | Controls (n=7) | MitoSNO (n=7) |
| --- | --- | --- |
| LVM (µl) | 120 ± 7 | 101 ± 6 * |
| LVEDV (µl) | 76 ± 6 | 60 ± 6 * |
| LVESV (µl) | 36 ± 4 | 22 ± 3 * |
| LVSV (µl) | 40 ± 3 | 38 ± 3 |
| LVEF (%) | 54 ±2 | 64 ±1 ** |
